# Experimental study on the explosion characteristics of hydrogen-methane premixed gas in complex pipe networks

**DOI:** 10.1038/s41598-021-00722-8

**Published:** 2021-10-27

**Authors:** Jinzhang Jia, Yinuo Chen, Guangbo Che, Jinchao Zhu, Fengxiao Wang, Peng Jia

**Affiliations:** 1 College of Safety Science and Engineering, Liaoning Technical University, Fuxin, 123000 Liaoning China; 2 Key Laboratory of Mine Thermo-Motive Disaster and Prevention, Ministry of Education, Huludao, 125105 Liaoning China; 3 Key Laboratory of Preparation and Applications of Environmental Friendly Materials (Jilin Normal University), Ministry of Education, Changchun, 130103 China

**Keywords:** Chemistry, Energy science and technology, Engineering

## Abstract

To explore the overpressure evolution laws and flame propagation characteristics in complex pipe networks after the addition of hydrogen to methane, we experimentally studied the explosive pressure wave and flame wave propagation laws for three different premixed gas mixtures with hydrogen-methane concentrations of 0, 10% and 20% when the equivalence ratio was 1. Experimental results indicate that the maximum explosion overpressure of the premixed gas increases with increasing distance from the explosion source, and it shows a gradually decreasing trend. In the complex pipe network, an overpressure zone is formed in the B–E–H and D–E sections of the network. The flame temperature is superimposed with the superimposition of the pressure, showing a trend of first increasing, then decreasing, then increasing, and finally decreasing in the complex pipe network. The flame arrival time increases with increasing distance, and the maximum flame speed shows a decreasing trend. The peak overpressure and maximum flame velocity of the premixed gas under a hydrogen volume fraction of 20% are 1.266 MPa and 168 m/s. The experimental research results could provide important theoretical guidelines for the prevention and control of fuel gas explosions in urban pipe networks.

## Introduction

As a clean, high-quality energy source, natural gas is widely used throughout many fields and for many purposes, and it is mainly transported through underground pipe networks. Methane is the main component of natural gas. In recent years, to extend the combustion limit of methane and increase the combustion rate, a suitable amount of hydrogen has been directly added to natural gas pipeline networks to enhance the reaction activity and increase the combustion temperature^1–4^. However, when the combustible gas leaks in the pipeline, hydrogen and methane mix with air to form a premixed gas. When the combustible gas and air mixture reaches the explosive concentration limit, it explodes when it encounters a fire source. Therefore, studying the propagation process of hydrogen-methane explosions in a complex pipe network, controlling the increase in pressure, and understanding the changing laws of flame propagation can effectively reduce the damage caused by explosions.

Many scholars have carried out theoretical and experimental research on the explosion propagation law of hydrogen addition to methane. Okafor et al.^5^ studied the propagation velocity of hydrogen-methane-air laminar premixed flames with different equivalent ratios and different volume fractions, and the results showed that under a certain equivalence ratio, the higher the hydrogen content in the mixture was, the faster the flame propagation speed. Halter et al.^6^ found through experiments that adding hydrogen to methane-air premixed gas increased flame stability and laminar flame velocity. Ma et al.^7,8^ studied the effects of added hydrogen on the characteristic parameters of methane-air explosion pressure and temperature through experiments and numerical simulations and found that adding hydrogen can effectively increase the explosive reaction activity and increase the explosion pressure and temperature. Woolley et al.^9^ established a mathematical model of the explosion pressure of gaseous hydrogen-methane-air mixtures, and the results showed that the addition of hydrogen can significantly increase the explosion pressure and temperature, thus increasing the explosion risk. Zhang et al.^10,11^ dynamically measured the explosion limits of methane-hydrogen mixtures with different components in a horizontal experimental pipeline. Zhou et al.^12^ conducted an experimental study on the explosion of premixed hydrogen and air in a confined space, obtained the explosion characteristics of the premixed gas and determined that the thin-walled strain response of the pipeline was in good agreement with the explosion pressure. In addition, the study of the pipeline structure on the flame propagation characteristics and dynamic behaviour of explosions is also of great significance for the prevention and control of flammable gas explosion disasters. Zhu et al.^13^ systematically studied the flame acceleration mechanism in pipe bends and bifurcation structures. Sulaiman et al.^14^ used FLACS numerical simulation software and found that the presence of a 90° turning structure would increase the flame speed by approximately 2 times. Emami et al.^15^ mixed hydrogen and air in 90° curved pipes and three-way pipes to conduct experiments to study the explosion characteristics, and the results showed that the mixed gas weakened the peak overpressure and flame propagation speed of the explosion in the curved pipe and that the peak overpressure and flame propagation speed of the explosion in the three-way pipeline were not affected. Zhu et al.^16^ carried out experimental research and a numerical simulation on the propagation characteristics of methane explosion flames and shock waves in a parallel structure network and determined that when the shock wave propagates in parallel pipes, the peak overpressure and maximum temperature continues to decrease. Niu et al.^17^ studied the propagation characteristics and attenuation laws of gas explosions in parallel network pipe networks, gradually complicating the pipeline structure.

Previous work on hydrogen-methane premixed gas explosions mainly focuses on the change characteristics of explosion overpressure and flame propagation velocity in straight pipes and simple structure pipelines. However, there have been only a few studies on the propagation laws of pressure waves and flame waves in complex networks^18–24^. The crisscrossing pipeline structure will make the propagation laws of pressure waves and flame waves more complicated, and only consider simple curves and bifurcations. It is not enough to consider only the propagation process in simple roadways such as bends and bifurcations. Therefore, a complex pipe network system was established to conduct a hydrogen-methane mixed explosion experiment in the current work. An experimental study was carried out to investigate the overpressure attenuation and flame propagation characteristics of premixed gases with three different hydrogen-methane concentrations when the equivalent ratio was 1. The aim was to determine the propagation law of hydrogen-methane explosions in a complex pipe network. The results of this work could serve as theoretical guidelines for the prevention and control of gas explosions in pipe networks.

## Pipe network experiment system

**Figure 1:**
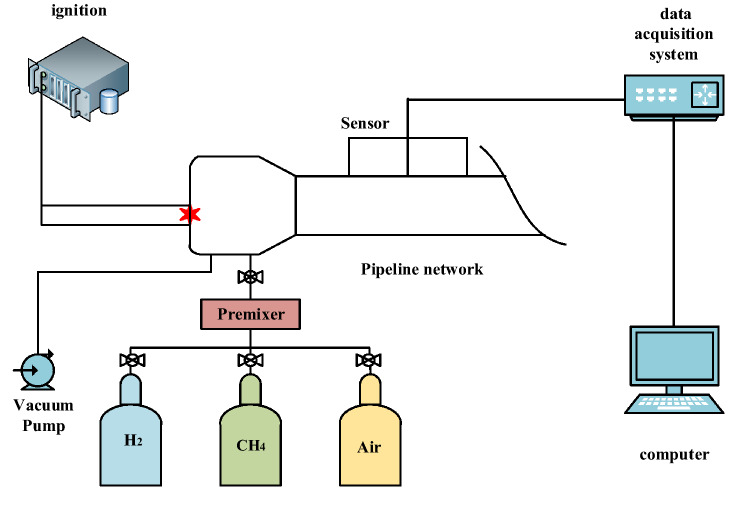
Experimental system.

The experimental device is shown in Fig. 1. The experimental pipeline system mainly includes 5 subsystems, namely, high-energy ignition device, gas distribution device, vacuum meter, explosion pipe network system, and dynamic data acquisition system. In the explosion pipe network system, the volume of the explosion chamber is 0.1 m^3^, the inner diameter of the cylindrical pipeline is 200 mm, and the wall thickness is 12 mm. The pipes in the system are all made of carbon steel, which is resistant to high temperature and corrosion and has a pressure resistance of more than 5 MPa. Each pipe includes a 20 mm diameter hole for inserting various sensors. The various components are connected with the pipes using internal threads. The accuracy of the experiments were maximized by increasing the air tightness of the pipeline network through the installation of a silicone gasket at the connection between each component and its corresponding pipe.

The ignition system mainly includes a high-energy igniter, a high-energy spark plug, a high-voltage and high-temperature-resistant cable, a power cable and an external trigger spark plug placed in the front of the explosion chamber, and a strong electric spark ignition is generated by alternating current with a voltage of 220 V and a frequency of 50HZ. The gas distribution system mainly uses three high-sensitivity mass flow controllers for direct gas distribution in accordance with the gas partial pressure law. The vacuum pump is used to send the prepared hydrogen-methane combustible gas into the gas filling area in the pipeline. Use the circulating pump to circulate the gas for 20 min, so as to ensure the uniform and full mixing of hydrogen, methane and air. An air compressor was used for 30 min of high-pressure ventilation after each experiment to discharge the residual exhaust gas from the explosion pipe network system. The TST6300 dynamic data acquisition and analysis system connects the dynamic data storage instrument, the pressure sensor, the flame sensor and the computer together.

**Figure 2:**
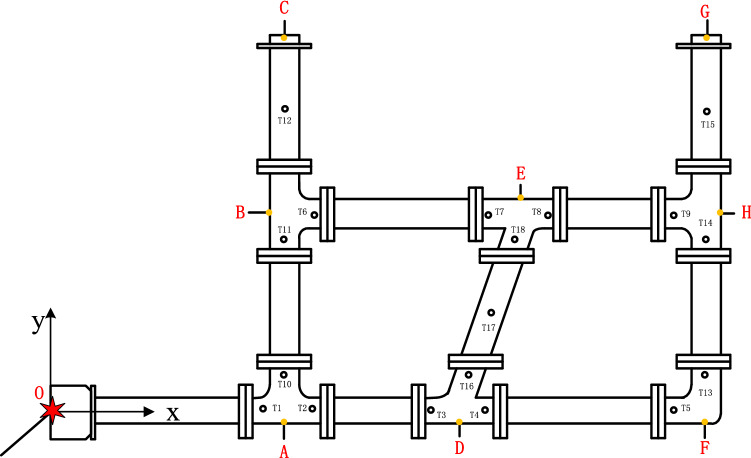
Transducer layout (Ti: flame and overpressure measured points).

In the network, a group of sensors are arranged along the pipe central line of each measuring point. Eighteen groups are arranged in total, and each group includes one pressure sensor, one temperature sensor and one flame sensor (O is the explosion source, A, B, D, E, and H are bifurcated structures, F is a turning structure, and C and G are pipe outlets). The arrangement of the sensor measuring points is shown in Fig. 2. Three tests are conducted for each experimental condition, and the numerical value obtained in the experiment is the mean of the three values. If point O is considered the origin of the coordinates, the direction O–A–D–F is the x-axis, and the direction A–B–C is the y-axis, Table 1 shows the coordinates of the measurement points and the explosion source.

**Table 1:** Location of each measured point from the explosion source (m).

Point	T1	T2	T3	T4	T5	T6	T7	T8	T9
Coordinate	(2.65,0)	(2.95,0)	(5.05,0)	(5.35,0)	(8.35,0)	(2.95,2.7)	(5.95,2.7)	(6.25,2.7)	(8.35,2.7)

Point	T10	T11	T12	T13	T14	T15	T16	T17	T18
Coordinate	(2.7,0.05)	(2.7,2.65)	(2.7,4.15)	(8.4,0.05)	(8.4,2.65)	(8.4,4.15)	(5.172,0.05)	(5.63,1.3)	(6.08,2.65)

For a multielement combustible gas mixture, its concentration can be expressed by the fuel equivalent ratio (*ψ*), which can be calculated by Eq. (1):

1 $$ \psi = \frac{{{F \mathord{\left/ {\vphantom {F A}} \right. \kern-\nulldelimiterspace} A}}}{{\left( {{F \mathord{\left/ {\vphantom {F A}} \right. \kern-\nulldelimiterspace} A}} \right)_{stoic} }} $$

where F/A is the fuel–air ratio and (F/A)_stoic_ is the fuel–air ratio at the stoichiometric concentration. *ψ* < 1 indicates a lean fuel mixture, *ψ* = 1 indicates a mixture at a stoichiometric concentration, and *ψ* > 1 indicates a rich fuel mixture.

An explosion experiment is carried out for the chemical dose concentration, that is, the CH_4_-H_2_ gas mixture under the condition of *ψ* = 1. The volume fraction of hydrogen in the mixed fuel is expressed as

2 $$ X = \frac{{V_{{{\text{H}}_{2} }} }}{{V_{{{\text{H}}_{2} }} + V_{{{\text{CH}}_{4} }} }} $$

where *V*_H2_ and *V*_CH4_ are the volumes of H_2_ and CH_4_ in the mixed fuel, respectively.

The experiment was carried out under ambient pressure (1.0 atm) and temperature (298 K). The main components are methane and hydrogen, and their purity is greater than 99.9%. Three premixed gases with concentrations of0, 10% and 20% were used (the equivalent ratio is 1).

The specific parameters are shown in Table 2.

**Table 2:** Compositions of hydrogen-methane-air mixtures.

No	Methane vol.%	Hydrogen vol.%	Air vol.%	Equivalence ratio *ψ*
1	9.5	0	90.5	1
2	6.31	10	83.69	1
3	3.84	20	76.16	1

## Experimental results and analysis

### Overpressure propagation laws of pressure waves in the pipe network

**Figure 3:**
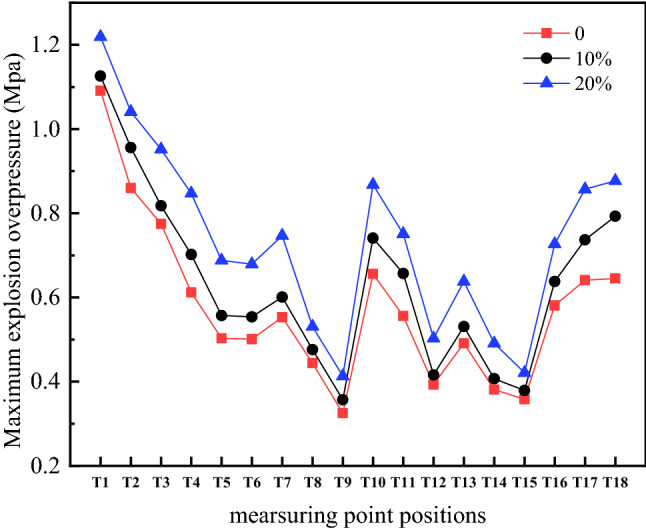
Maximum explosion overpressure of all measuring points.

Figure 3 shows the maximum explosion overpressure of the hydrogen-methane pressure wave propagating in the complex pipe network under three hydrogen volume fractions. As shown in the figure, when the volume fraction of hydrogen is 20%, the maximum explosion overpressure of the premixed gas is higher than the volume fractions of 10% and 0. The maximum explosive overpressure of the premixed gas increases as the volume fraction of added hydrogen increases. When the volume fraction of hydrogen is less than 20%, the explosion intensity is reduced. In the complex pipe network, the explosion overpressure at the T1 measuring point at bifurcation structure A reaches the highest, and the explosion overpressure at the T15 measuring point at pipe outlet G attenuates to the lowest.

**Figure 4:**
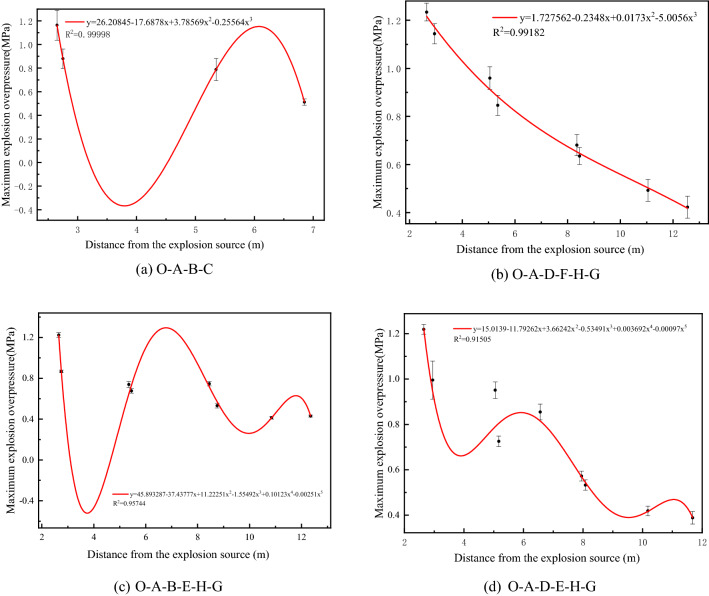
Maximum explosion overpressure for a 20% hydrogen volume fraction. (**a**) O–A–B–C branch; (**b**) O–A–D–F–H–G branch; (**c**) O–A–B–E–H–G branch; (**d**) O–A–D–E–H–G branch.

Figure 4 shows the relationship between the maximum explosion overpressure and distance in the O–A–B–C, O–A–D–F–H–G, O–A–B–E–H–G and O–A–D–E–H–G branches under a hydrogen volume fraction of 20%. The premixed gas reaches the maximum explosion overpressure peak at the T1 measurement point in the O–A–B–C, O–A–D–F–H–G, O–A–B–E–H–G and O–A–D–E–H–G branches and then passes through the bifurcation and turning structures. The explosion overpressure continues to decrease, and due to the combined effect of combustible gas consumption, pressure wave energy loss, and pipe wall heat dissipation, measurement points T12 and T15 at pipe outlets C and G attenuate to a minimum. Fitting the maximum explosion overpressure and distance in the O–A–B–C, O–A–D–F–H–G, O–A–B–E–H–G and O–A–D–E–H–G branches shows that the maximum explosion overpressure in the four branches gradually decreases as the distance from the explosion source increases. However, the R^2^ values in the O–A–B–E–H–G and O–A–D–E–H–G branches are 0.95744 and 0.91505, respectively, which are larger than the R^2^ values in the O–A–B–C and O–A–D–F–H–G branches. This is because the B–E–H and D–E sections of the O–A–B–E–H–G and O–A–D–E–H–G branches are in the middle of the complex pipe network, which easily withstands repeated shocks in different directions of overpressure in different branches of the pipe network. In branches B–E–H and D–E, the maximum explosion overpressure of the measuring point increases, which leads to greater error in the fitted results.

**Figure 5:**
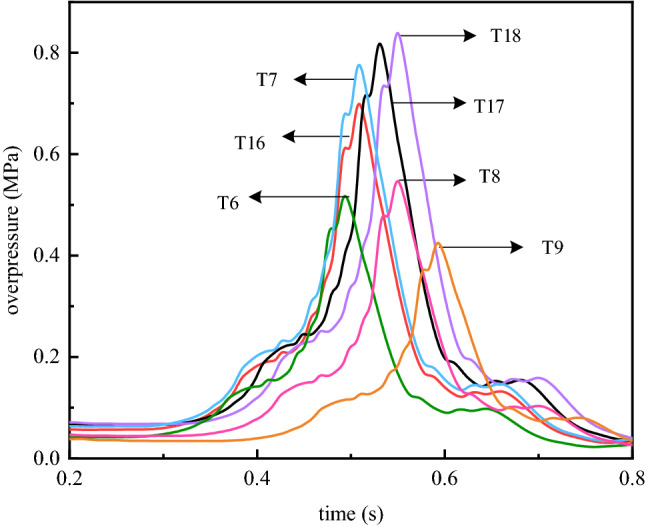
Maximum explosion overpressure at the measuring point in the middle of the complex pipe network.

Figure 5 shows the curve of the maximum explosion overpressure at some measuring points of the complicated pipe network with time under a hydrogen volume fraction of 20%. When the pressure wave of the premixed gas propagates to measuring point 18, the maximum explosion overpressure is approximately 0.877 MPa, which is 18.2% and 2.6% higher than those of measuring point T16 and measuring point T18, respectively. Compared with measuring point 16, the pressure of measuring point 17 is increased by approximately 16.8%. The maximum explosion overpressure near the centre of the B–E–H branch gradually increases, forming an enlarged area. This result is mainly due to the appearance of opposing pressure waves in the B–E–H branch. When pressure waves from the A–B–C branch and the F–H–G branch, which are opposite to each other, meet in the B–E–H branch, the oscillating pressure waves are superimposed, causing the pressure to rise. Similarly, the pressure wave of the premixed gas explosion propagates to measurement point T7 in the middle of the pipe network. The maximum explosion overpressure is approximately 0.745 MPa. Compared with measuring points T6, T8 and T9, the maximum explosion overpressure increases by approximately 10.1%, 28.9% and 44.7%, respectively. The overpressure gradually increases near the centre of the D–E branch, and an area of increase is also formed. This is because pressure waves from the B–E–H branch direction and the opposite direction of the A–D–F branch appear in the D–E branch. When they meet in the D–E branch, the pressure waves are superimposed, and the pressure rises. Although the time for the pressure wave to propagate to measuring point T7 is shorter than that of measuring point T18, the overpressure at measuring point T18 is higher than the overpressure at measuring point T7. This is because although the energy is shunted many times, most of it is still spread throughout the main straight pipe. At the same time, measuring point T18 accumulates the energy from measuring points T17, T7 and T8. Because branches B–E–H and D–E are located in the middle of the complex pipe network, they easily withstand the repeated oscillations of overpressure in different branches of the pipe network. Under the action of the reverse pressure wave, a high-pressure area with strong destructive power is formed in the middle of the pipe network.

### Flame propagation laws of pressure waves in the pipe network

**Figure 6:**
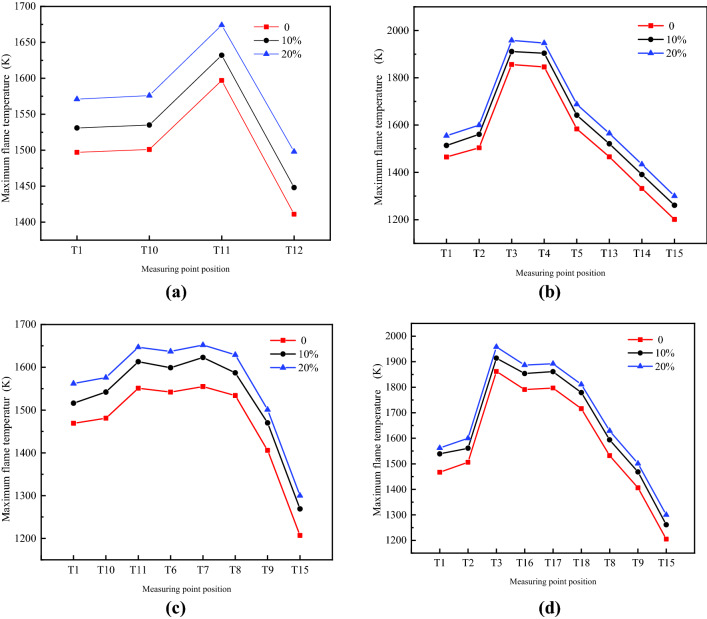
Maximum flame temperature of all measuring points. (**a**) O–A–B–C branch; (**b**) O–A–D–F–H–G branch; (**c**) O–A–B–E–H–G branch; (**d**) O–A–D–E–H–G branch.

Figure 6 shows the maximum flame temperature of the measuring points in the O–A–B–C, O–A–D–F–H–G, O–A–B–E–H–G and O–A–D–E–H–G branches under three hydrogen volume fractions. As shown in the figure, at the maximum temperature of each measuring point in the branch, the mixture with the hydrogen volume fraction of 20% has a higher temperature than 10% mixture and the sample that does not contain hydrogen. When the hydrogen volume fraction is 20%, the temperature in the O–A–B–C branch is1555 K at T1; it then rises slowly to 1587 K at T10, reaches the highest point of 1647 K at T11, and finally drops. The flame temperature of other branches also increase first and then decrease. However, in the O–A–B–E–H–G and O–A–D–E–H–G branches, T3 and T11 first reach their peaks, then fall, and then reach their second peaks at T7 and T17, respectively, and then fall again. The overall flame temperature has a trend of repeatedly rising and falling. This trend occurs because the flame temperature of the B–E–H and D–E branches entering the middle of the pipe network increases. This result is due to the gradual increase in the expansion of the gas during the flame propagation process through the bifurcation and turning, the gas causes the disturbance to increase the flame surface, and the negative feedback effect of the compression wave on the flame propagation during the flame propagation makes the flame in the tube a backflow phenomenon has occurred, resulting in a new peak in the flame temperature in the B–E–H and D–E branches. Then, as the propagation distance increases, the maximum flame temperature at pipe outlet G decreases, and the flame attenuation is more obvious. The flame temperature during the explosion propagation process of hydrogen-methane premixed gas in the complex pipe network space shows a trend of first increasing, then decreasing, then increasing, and then decreasing.

**Figure 7:**
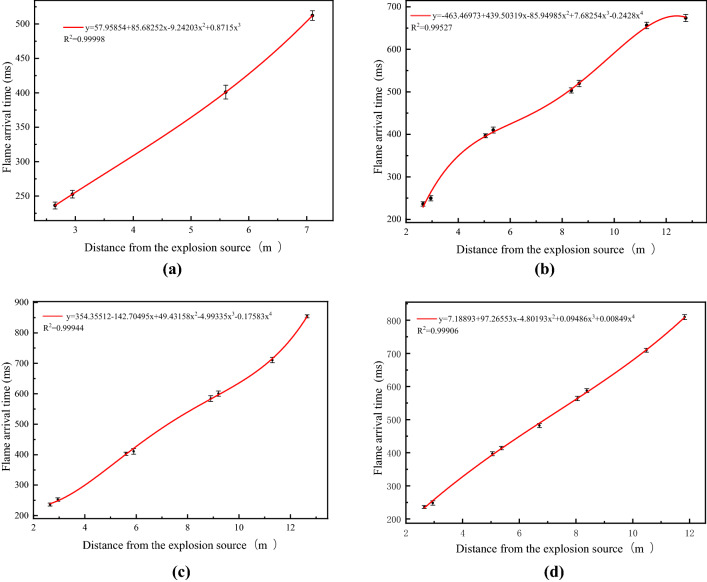
Flame arrival time for 20%. (**a**) O–A–B–C branch; (**b**) O–A–D–F–H–G branch; (**c**) O–A–B–E–H–G branch; (**d**) O–A–D–E–H–G branch.

Figure 7 shows the flame arrival time at the measuring points in the O–A–B–C, O–A–D–F–H–G, O–A–B–E–H–G and O–A–D–E–H–G branches under a hydrogen volume of 20%. Fitting the flame arrival time and distance to obtain the formula shows that as the distance from the explosion source increases, the flame arrival time gradually increases.

To analyse the flame propagation velocity in the pipe network, we calculate the flame propagation velocity with Eq. (3):

3 $$ v = \frac{{x_{n} }}{{t_{n + 1} - t_{n} }} $$

where *v* is the flame propagation velocity, *x*_*n*_ is the distance from the n + 1-th flame sensor to the n-th flame sensor, *t*_n+1_ is the time for the n + 1-th flame front end to arrive at the flame sensor, and *t*_n_ is the time for the n-th flame front end to arrive at the flame sensor.

**Figure 8:**
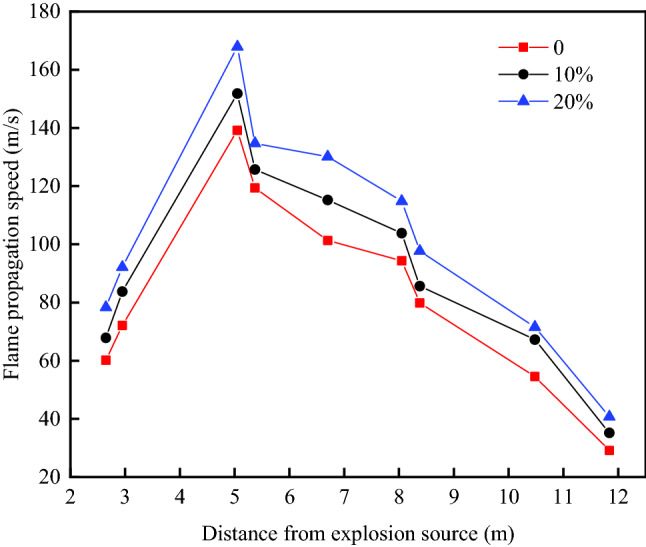
The maximum flame propagation velocity of the O–A–D–E–H–G branch.

Figure 8 shows the flame propagation velocity distribution in the O–A–D–E–H–G branch under three hydrogen volume fractions. The velocity of hydrogen-methane premixed gas shows an upward and downward trend after the explosion. When the volume fraction of hydrogen is 20%, the pressure wave generated by the explosion breaks through the film. Under the action of high temperature and high pressure, the hydrogen-methane gas reacts fully with oxygen, and the flame begins to accelerate. The flame velocity increases from 78.3 m/s at T1to 92.1 m/s at T2. In the initial explosion stage, the pressure wave propagates in the straight pipe, and the flame propagates slowly. The maximum velocity rise at T3 is 167.9 m/s, and the flame propagation noticeably accelerates because the reaction is intensified under the guidance of turbulence, the flame front expands rapidly after bifurcation and turning and propagates towards different branches. After passing through measuring point T3, the flame propagation begins to decelerate due to reasons such as insufficient fuel, wall reflection and pipe heat dissipation, making the maximum flame propagation velocity continuously decrease in each branch. By the time the flame reaches measuring point T15 of pipe outlet G, the flame propagation velocity has been reduced to its minimum. The flame propagation velocity is higher under a hydrogen volume fraction of 20%than under a 0% or 10% hydrogen volume fraction, and the velocities in the other branches are similar.

From the perspective of chemical reaction kinetics, hydrogen (H_2_) has a larger C–H bond energy than methane (CH_4_). As a result, the burning rate of a single gas is slow, the flame propagation velocity is low and the combustion is incomplete at low concentrations, and the activity of hydrogen is high. Adding a little hydrogen to methane will have a great impact on the overall properties of the premixed gas, and the proportion of hydrogen will increase. High enhances the concentration of energy release. The main reason for the flame propagation velocity of hydrogen-methane-air mixed gas is the free radical content of CH_4_ combustion in the gas. The mixing of H_2_ promotes the flame reaction to a certain extent^25^. The detailed elementary reaction model of methane combustion is GRI3.0. Mechanism (53 components, 325 elementary reactions). The elementary reactions related to the main generation and consumption of CH_4_ are:

$$ \begin{aligned} & {\text{OH + CH}}_{{4}} \leftrightarrow {\text{CH}}_{{3}} {\text{ + H}}_{{2}} {\text{O}}\quad {\text{(R98)}} \\ & {\text{H + CH}}_{{4}} \leftrightarrow {\text{CH}}_{{3}} + {\text{H}}_{{2}} \quad {\text{(R53)}} \\ & {\text{O}} + {\text{CH}}_{{4}} \leftrightarrow {\text{OH + CH}}_{{3}} \quad {\text{(R11)}} \\ & {\text{H}} + {\text{CH}}_{{3}} {\text{( + M)}} \leftrightarrow 4{\text{( + M)}}\quad {\text{(R52)}} \\ \end{aligned} $$

The elementary reaction “${\text{H + O}}_{{2}} \leftrightarrow {\text{O + OH}}\quad {\text{(R38)}}$” is the most sensitive of all the elementary reactions^25^. The addition of more hydrogen significantly increases the forward reaction rate^26^; therefore, significantly extends the combustion limit of methane and increases the combustion rate and flame propagation velocity.

### Overpressure attenuation and flame mutation in a complex pipe network

Under these three hydrogen volume fractions, the change in the peak overpressure of the explosion wave of each structure in the pipe network is expressed by the shock wave peak overpressure attenuation factor *μ*, and the calculation of *μ* is shown in Eq. (4):

4 $$ \mu = \frac{P}{{P^{\prime } }} $$

where *P* is the peak overpressure of the explosion wave before the bifurcation or turning structure in units of MPa; *P*′ is the peak overpressure of the explosion wave after the bifurcation or turning structure of the pipe in units of MPa; *μ* is a dimensionless quantity.

To calculate the change in the flame velocity of the pipes of each structure in the pipe network for the mixtures with the three different hydrogen ratios, *ε* is used to represent the mutation rate of the peak size of the flame propagation velocity. The calculation of *ε* is shown in Eq. (5):

5 $$ \varepsilon = \frac{v}{{v^{\prime } }} $$

where *v* is the flame propagation velocity before the bifurcation or turning structure; *v*′ is the flame propagation velocity after the bifurcation or turning structure; and *ε* is a dimensionless quantity.

The experimental pipe network includes 5 bifurcation structures and 1 turning structure. Equation (4) and Eq. (5) are used to calculate the maximum explosion overpressure and flame propagation velocity changes at 11 locations in the pipe network.

**Figure 9:**
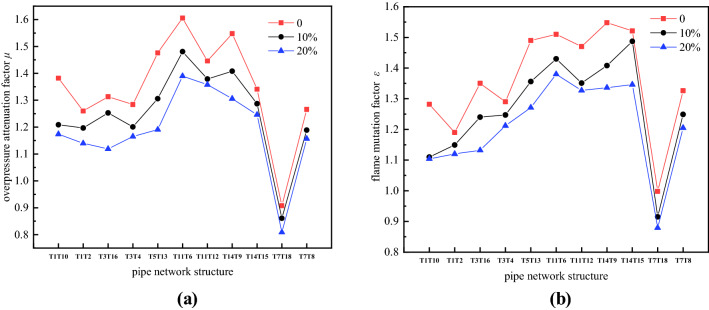
Overpressure attenuation factor and flame mutation factor of the pipe network. (**a**) *μ*; (**b**) *ε*.

Figure 9a shows the overpressure attenuation factor of the pipe network. It can be observed that adding hydrogen to methane can reduce the attenuation of methane explosions in the pipe network. This is because when the higher sensitivity hydrogen is mixed into the lower sensitivity methane-oxygen premix, the entire premixed gas sensitivity increases. Compared with the case where hydrogen is not added, the highly sensitive gas has greater instability after the addition of hydrogen during the explosion near the pipe outlet. The movement is more violent and is relatively less affected by the expansion wave. Therefore, the explosion intensity near the bifurcation and turning structures of the pipe network are less attenuated after the addition of hydrogen. Figure 9b shows the flame mutation factor in the pipe network. When the volume fraction of added hydrogen increases, the effect of the premixed gas flame mutation near the bifurcation and turning structures of the pipe network are weakened.

**Figure 10:**
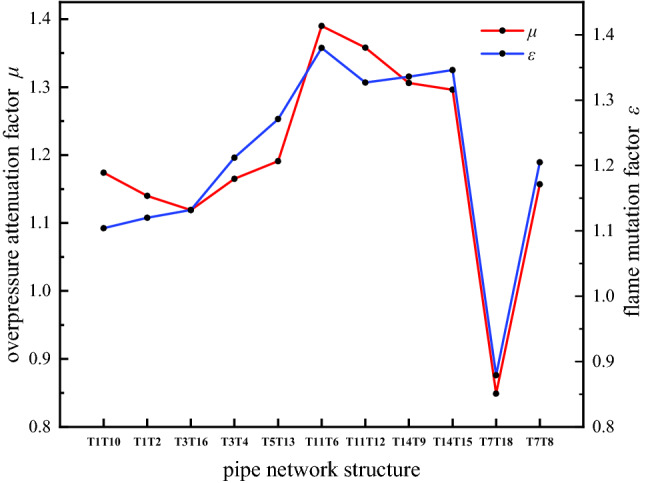
Pipe structure inhibition of pressure and flame.

Figure 10 shows the influence of the structure of the complex pipe network on the overpressure attenuation and flame mutation. The pressure attenuation factor and flame mutation factor at the bifurcation structure of B and H in the pipe network in this experiment are relatively large, which means that the pressure and flame decrease greatly after the branch flow of the bifurcation structure. The pressure attenuation factors and flame mutation factors at the D and E bifurcation structures are relatively small, which means that the pressure and flame decrease after the split flow through the bifurcation is small. Although the energy generated by the explosion propagates to the bifurcation structure of the pipeline, its propagation direction and magnitude change, most of the energy is concentrated in branches O–A–B–C and O–A–D–F–H–G of the pipe network, and the energy entering the middle of the pipe network B–E–H and D–E branches is reduced, weakening the explosion to a certain extent. However, due to the influence of the pipe network geometry, the energy meets in the opposite direction in the B–E–H and D–E branches so that the pressure and temperature increase instead of decrease. Therefore, under the repeated action of opposite energy waves in different routes, a high-temperature and high-pressure zone is formed in the middle of the pipeline, and the destructive force increases. The geometric structure of the pipe network is an important factor that affects the attenuation of the hydrogen-methane premixed gas explosion energy in the pipe network.

## Conclusions

1. After different hydrogen volume fractions are added to an explosion of methane in a pipe network, the maximum explosion overpressure shows an increasing trend with increasing hydrogen volume fraction. The premixed gas with a hydrogen volume fraction of 20% has the most complete reaction, produces a stronger pressure wave, and has a faster flame propagation speed. At 1.266 MPa and 168.7 m/s, the maximum explosion overpressure and flame propagation speed are the largest, respectively.
2. After the hydrogen-methane premixed gas explodes in the pipe network, the maximum explosion overpressure of the premixed gas increases with increasing distance from the explosion source in the four branches of the complex pipe network, and the maximum explosion overpressure shows a gradual decreasing trend. The B–E–H and D–E sections of the O–A–B–E–H–G and O–A–D–E–H–G branches of the complex pipe network easily withstand repeated oscillations in different directions of overpressure in different branches of the pipe network, resulting in superimposition of pressure waves and the formation of high-pressure areas in the B–E–H and D–E branches; the superimposed pressure produces strong explosive impact and destructive force.
3. After the hydrogen-methane premixed gas explodes in the pipe network, the maximum flame temperature first increases and then decreases. Due to the reverse pressure wave and the subsequent forward pressure wave in the B–E–H and D–E branches, the temperature of the flame increases again and eventually decreases. The flame arrival time increases with increasing distance. The maximum flame propagation speed first rises and then gradually decreases. Near pipe outlet G, the flame speed decays to its lowest value.
4. The overpressure attenuation factor and flame mutation factor of the explosion at the bifurcation and turning structures in the pipe network increase the sensitivity of the premixed gas due to the increase in the hydrogen volume fraction. The increased sensitivity affects the explosion overpressure attenuation and flame mutation, resulting in a slow attenuation of the explosion intensity. In addition, due to the geometrical structure of the pipeline, the opposite energy waves of different paths repeatedly act on the B–E–H and D–E branches in the middle of the complex pipe network to form a high-temperature and high-pressure zone, which increase the destructive power.

## Data Availability

The datasets generated and analyzed during the current study are available from the corresponding author on reasonable request.
